# Age- and Gender-Specific Reference Values for Physical Performance in Tunisian Youth Basketball Players

**DOI:** 10.3390/children11111346

**Published:** 2024-11-01

**Authors:** Chirine Aouichaoui, Mohamed Tounsi, Ghazi Racil, Oussama Tabka, Monia Zaouali, Nicola Luigi Bragazzi, Yassine Trabelsi

**Affiliations:** 1 Research Laboratory, Exercise Physiology and Physiopathology: From Integrated to Molecular “Biology, Medicine and Health”, LR19ES09, Faculty of Medicine of Sousse, Sousse University, Sousse 4000, Tunisia; chirineaouichaoui@yahoo.com (C.A.); m.tounsi@hotmail.fr (M.T.); oussamatabka@hotmail.fr (O.T.); zaoualimonia@yahoo.fr (M.Z.); trabelsiyassine@yahoo.fr (Y.T.); 2 High Institute of Sport and Physical Education of Ksar Saïd, University of Manouba, Manouba 2010, Tunisia; 3 Sport Performance, Health & Society, Higher Institute of Sport and Physical Education of Ksar Said, Manouba 2010, Tunisia; ghazi_racil@yahoo.fr; 4 Department of Mathematics and Statistics, Laboratory for Industrial and Applied Mathematics (LIAM), York University, Toronto, ON M3J 1P3, Canada; 5 Human Nutrition Unit (HNU), Department of Food and Drugs, Medical School, Building C, Via Volturno, 39, 43125 Parma, Italy

**Keywords:** performance parameters, body measurement, reference values, basketball players

## Abstract

Background/Objectives: Physical performance is becoming increasingly critical in basketball, as it directly influences players’ agility, power, and endurance. This study aimed to assess the progression of body composition and physical performance metrics across different ages and genders, establishing age- and gender-specific reference values for Tunisian basketball athletes. Methods: A total of 469 Tunisian basketball players (239 boys and 230 girls) were assessed and grouped by age. Anthropometric measures—including standing and sitting height, body mass, leg length, body mass index, fat mass, fat-free mass, body fat percentage, wingspan, and leg muscle volume—were collected alongside physical performance metrics. Performance tests included countermovement and squat jumps, change-of-direction speed, maximal oxygen uptake, flexibility, the five-jump test, and 5 m, 10 m, and 20 m sprints with and without the ball. Normative data were generated based on age and gender categories. Results: The findings revealed significant age-related improvements in both anthropometric and performance parameters. Boys consistently outperformed girls in physical and fitness-related measures, with gender differences becoming more pronounced with age. Stepwise regression analyses indicated that, for boys, body fat percentage, leg muscle volume, standing height, and wingspan were the best predictors of physical performance. For girls, body fat percentage, standing height, and sitting height were identified as key predictors. Conclusions: The newly established Tunisian reference values for physical performance in youth basketball provide valuable benchmarks that can support the development of explosive power and strength in players, aiding in talent identification and potentially enhancing individual and team performance outcomes.

## 1. Introduction

Basketball is a highly popular, high-intensity sport that demands a range of skills, such as jumping, sprinting, and sprinting with changes of direction (CODs) [1]. Executing these movements during gameplay engages both the anaerobic and aerobic energy systems. A well-developed endurance capacity is essential for athletes, as it enhances their ability to recover more efficiently during a match [2].

In basketball, performance is multifaceted, involving an integration of anthropometric, physiological, psychological, tactical, and technical attributes, all of which play a crucial role in determining player success. In addition to technical–tactical skills [3], body measurements and physiological metrics are pivotal in evaluating player success across all age groups [4]. Physical characteristics, including agility, endurance, power, strength, and speed in both the upper and lower limbs, are integral. Muscle strength, in particular, serves as the foundation upon which performance in basketball is built [5], with significant improvements observable during middle-to-late childhood. This improvement, however, is influenced by various factors such as gender, fitness level, body growth, somatic maturation, and motor skills [4].

Vertical jump performance is a critical indicator of lower limb strength and, therefore, is frequently used as a valuable measure of athletic performance across sports. In basketball specifically, a player’s vertical jump is directly tied to their on-court performance. The anthropometric profile of players can be a limiting factor in their performance, and the National Basketball Association (NBA) underscores the importance of physical profiling in the selection process [6].

Anthropometric characteristics, including factors like body fat percentage, height, wingspan, and circumferential measurements, are recognized as significant parameters in elite basketball athletes [7]. Other studies have also shown that these anthropometric and physical traits predict success in young players and aid in identifying talent [7]. However, determining the correlation between specific physical and anthropometric variables and player success remains challenging. Moreover, an important research question is how these variables can reliably inform predictive models for player performance.

Sansone et al. [8] conducted a study providing normative values for body fat mass in basketball athletes, considering measurement methods, gender, and competition level. However, research investigating the relationship between anthropometric and physical performance parameters in basketball is limited [9]. Moreover, understanding these relationships across age groups is essential for enhancing player performance. Rismayadi et al. [10] reported differences in physical conditions among 16- to 18-year-old players in various positions (guards, forwards, and centers) [10].

In Chile, Hernandez-Martinez et al. [11] found that male athletes with higher fat-free mass and lower body fat percentages performed better in the countermovement jump (CMJ), ball throwing, and maximal isometric handgrip strength tests [11]. Previous studies have demonstrated significant differences in physical test outcomes according to age, gender, and playing position [12,13], yet no predictive relationship was established between performance indicators and anthropometric variables. Recently, Čaušević et al. [14] showed that anthropometric factors could predict agility and speed profiling among U14, U15, and U16 basketball players in Sarajevo Canton, examining the association between physical parameters, anthropometric profiles, and body composition profiles [14].

In Tunisia, data on the predictive parameters of physical performance in basketball players is scarce. Understanding predictive physical variables, considering gender and age, is crucial for Tunisian basketball coaches, allowing them to tailor training programs effectively and enhance performance. Statistical models are required to provide coaches with a robust tool for assessing performance and identifying talent efficiently. In this context, the present research was conducted with the hypothesis that basketball talent identification and game performance indicators could be predicted from anthropometric and physical tests.

The objectives of this study were (i) to evaluate the progression of morphological parameters and performance metrics across age and gender, (ii) to identify the anthropometric parameters with the highest predictive value for performance in jumping and speed tests among Tunisian players in the U13, U15, U17, and U20 age categories, and (iii) to establish reference values for physical performance in youth basketball players.

## 2. Materials and Methods

### 2.1. Participants

Four hundred and sixty-nine Tunisian basketball players (239 boys and 230 girls) were randomly chosen from various basketball teams. Their mean weight, height, and age were 62.59 ± 14.32 kg, 169.81 ± 12.87 cm, and 15.27 ± 2.44 years, respectively. Before conducting the study, both players and their guardians were fully informed of the research objectives and procedures and signed a written informed consent form. All athletes were evaluated by a physician and confirmed to be in good health. During the competitive season, players participated in the championship league on a weekly basis. They had a similar training volume, totaling 8 h and 40 min per week, which included three technical sessions (1.5 h each), three conditioning and strength sessions (40 min each), and two hours of gameplay. Players abstained from exercise the day before testing and avoided caffeine beverages for four hours prior to testing.

On three separate days, players completed physical performance tests, which included the squat jump (SJ), CMJ, five-jump test, speed, agility, flexibility, and aerobic shuttle run. Participants were instructed to avoid intense training, alcohol, and tobacco use for two days before testing. Only water was allowed up to two hours before each test, and no food was allowed three hours before testing. Each test session began with a standardized 15 to 20 min warm-up. The intraclass correlation coefficients (ICCs) for the test–retest reliability of the study’s variables were greater than 0.95, with the coefficients of variation (CVs) ranging from 0.9% to 8.1%.

The research protocol was approved by the Research Ethics Committee of the Faculty of Medicine of Sousse, Tunisia (CEFMS: 213/2023). The study adhered to the ethical standards of the World Medical Association’s Declaration of Helsinki of 1964, as amended in 2013, and received local institutional review board approval [15].

### 2.2. Anthropometric Measurements

Sitting and standing heights were measured using a Harpenden Portable Stadiometer (Harpenden, UK). Body mass was recorded with a digital Harpenden Balance Scale (UK). Leg length was calculated as the difference between standing and sitting heights [16]. Body mass index (BMI) was derived by dividing body mass (kg) by the square of standing height (m).

Lower body impedance was assessed with a Tanita TBF-604 Body Fat Monitor/Scale (Tokyo, Japan), which requires input parameters such as the body mass, standing height, and gender of the participant. The device measures lower body impedance and calculates body fat percentage [17]. Absolute body fat mass was then calculated using the following formula: fat mass (kg) = (percentage body fat × body mass)/100. Fat-free mass (kg) was determined by subtracting fat mass from total body mass.

Wingspan was measured with a tape measure from fingertip to fingertip, with the subject standing upright against a wall and arms extended horizontally.

The volume of leg muscle in the dominant leg was determined using anthropometric methods, as outlined by Jones and Pearson [18]. Seven circumference measurements were taken at specific sites: the maximum gluteal furrow, maximum mid-thigh, minimum above the knee, maximum knee, minimum below the knee, maximum calf, and minimum ankle. The heights of these circumferences from the floor were also recorded. Additionally, skinfold thickness measurements were obtained at the thigh (second circumference) and calf (sixth circumference) sites [18].

### 2.3. Vertical and Horizontal Jump Assessments

Vertical jump performance was assessed using an Optojump device (Microgate SRL, Bolzano, Italy) connected to a portable computer for data collection. This setup captured metrics including jump height, power, flight time, and contact time. Two types of vertical jump tests were performed: the SJ and the CMJ. Athletes were instructed to perform two practice jumps, with the best performance from three subsequent trials recorded as the final result. A two-minute rest period was provided between each jump to ensure adequate recovery [19].

For the SJ test, athletes descended to and held a knee angle of approximately 90 degrees for three seconds before jumping as high as possible, without any preliminary countermovement. A valid trial required no downward movement prior to the jump [19].

In the CMJ test, athletes started from a standing position, quickly descending before jumping as high as possible in the concentric phase. Verbal encouragement was provided to maximize jump height [19]. Both types of jumps were performed without the use of arms. Each participant completed three attempts for each jump type, with the highest value from each type used for analysis. Athletes wore comfortable clothing and running shoes during the tests. The CV for the reliability of this test was 3.2%, with a 95% confidence interval (CI) of 0.98–0.99.

The five-jump test was also conducted to assess explosive leg strength and measure the distance covered. Athletes began the test by standing with their feet together, choosing their lead foot for the initial jump. They were instructed to complete the final stride with feet together. Distance covered was measured with a tape measure [20].

### 2.4. Sprint Tests

Speed assessments involved 5 m, 10 m, and 20 m sprints, conducted both with and without the ball. Prior to testing, participants completed a standardized warm-up routine, beginning with a light jog, followed by dynamic exercises targeting flexibility in muscle groups critical for sprinting [21]. The players’ performance was measured using the Witty timing system by Microgate. Participants started each sprint from a stationary position, positioned half a meter behind the starting line. Each player completed three trials per distance, with a minimum recovery period of five minutes between attempts. The fastest recorded time, measured to the nearest 0.01 s, was taken as the player’s speed score. Verbal encouragement was provided to ensure maximal effort in each sprint [21].

### 2.5. Flexibility

Flexibility was assessed using an anteflexion meter (TKK-5403) to measure hamstring and lower back flexibility [22]. This digital device records the degree of forward body flexion, with a measuring range from −20.0 to +35.0 cm. During testing, players stood barefoot on a platform with their legs straight and together, then bent forward, reaching as far as possible to gauge maximum flexibility [22].

### 2.6. Modified Agility T-Test (MATT)

The modified agility T-test (MATT) is a running assessment that evaluates changes in direction speed through various techniques. Agility timing was measured with the Microgate portable timing system. The MATT followed the standard T-test protocol, with adjustments to the total distance covered and the spacing between cones. Participants were verbally encouraged to perform multidirectional movements as quickly as possible, typically resulting in approximately 45-degree CODs. The best performance from the final two trials in the reliability assessment session was recorded as the agility score [23].

### 2.7. Aerobic Power Test

Aerobic capacity, based on maximal aerobic speed, was assessed using the 20 m maximal multistage run test, initially developed by Léger and Lambert [24] and later modified by Léger et al. [25]. In this test, participants sprinted back and forth between two lines set 20 m apart, with continuous encouragement to sustain their effort for as long as possible. The pace was dictated by a cassette tape emitting timed beeps, beginning at 8.5 km per hour for the first minute and increasing by 0.5 km per hour each minute thereafter. The test concluded when participants could no longer maintain the required pace. The number of completed shuttles was recorded for further analysis [25].

Data from the final stage enabled the calculation of each participant’s maximal oxygen uptake (VO_2_ max), expressed in mL.kg^−1^.min^−1^. Maximal Aerobic Speed (MAS) was defined as the minimum speed at which participants achieved their VO_2_ max, representing their peak aerobic power level [25].

### 2.8. Statistical Analysis

Descriptive statistics were calculated, with all measurements reported as the mean ± standard deviation (SD). The normality of the data was assessed using the Kolmogorov–Smirnov test and confirmed with Q-Q plots. Differences in body size and performance parameters across age categories and gender were analyzed using multivariate analysis of variance (MANOVA), with a Bonferroni correction applied to account for multiple comparisons. Partial eta squared (η^2^) values from the MANOVA results were used to determine effect sizes, categorized as follows: no effect (0 to 0.039), minimum (0.04 to 0.24), moderate (0.25 to 0.63), and strong (≥0.64) [26]. Multiple regression analyses were conducted to examine the relationships between anthropometric parameters, jumping performance, and sprint performance, with separate models computed for boys and girls according to age groups. All statistical analyses were performed using the commercial software ‘‘Statistical Package for the Social Sciences’’ (SPSS version 26.0, IBM, Armonk, NY, USA), with a significance level set at *p* < 0.05.

## 3. Results

### 3.1. Anthropometry and Physical Performances Differences Based on Age Groups and Gender

**Figure 1:**
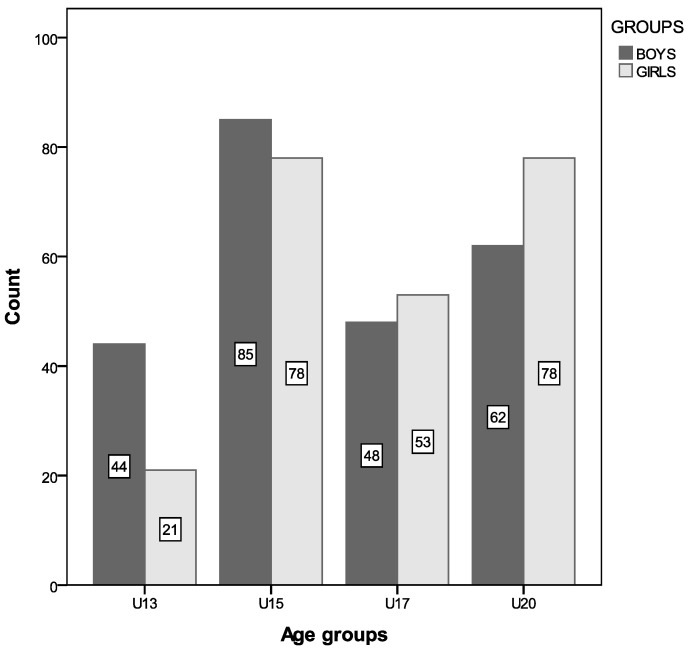
Distribution of Tunisian basketball players according to age categories and gender.

The distribution of Tunisian basketball players based on age categories and gender is displayed in Figure 1.

A total of 469 Tunisian basketball athletes (239 boys and 230 girls) participated in this study. Descriptive statistics for anthropometric parameters are summarized in Table 1, while the mean ± standard deviation for physiological and physical performance measures are provided in Table 2. Across all participants, there was an increase in anthropometric and physiological parameters with chronological age, regardless of gender, except for sprint and agility times, which decreased with age (as shown in Table 1 and Table 2). Significant differences (*p* < 0.05) between genders emerged from the U15 category onward, with boys being significantly heavier and taller than girls (see Table 1). Additionally, boys demonstrated superior physical and physiological performance compared to girls (Table 2). Notably, jump heights in the CMJ test were significantly higher (*p* < 0.05) than those in the SJ test for both boys and girls (as indicated in Table 2).

### 3.2. MANOVA Analyses Evaluating Difference in Anthropometric and Physical Performance Parameters by Age Categories and Gender

The MANOVA results, including the main and interaction effects as well as effect sizes, are presented in Table 3 and Table 4. These results examine differences in anthropometric and performance parameters across age categories, gender, and test batteries.

#### 3.2.1. Interaction Effect of Gender and Age

Our findings revealed significant age × gender interaction effects for most anthropometric variables, with the exception of BMI, body fat percentage, and leg muscle volume (see Table 3). Additionally, significant age × gender interactions were observed for vertical jumps, the five-jump test, 5 m sprints, and 10 m sprints (*p* < 0.05) (Table 3). For anthropometric variables, Wilks’ Lambda for the age category × gender interaction was Λ = 0.756, F(30, 1327) = 4.427, *p* < 0.01, η^2^ = 0.089. For physical performance variables, Wilks’ Lambda for the age category × gender interaction was Λ = 0.589, F(51, 1316) = 5.019, *p* < 0.01, η^2^ = 0.162.

#### 3.2.2. Effects of Chronological Age

Significant main effects of chronological age were observed across all anthropometric parameters (*p* < 0.01; η^2^ ranging from 0.035 to 0.399). For anthropometric measures by age category, Wilks’ Lambda was Λ = 0.499, F(30, 1327) = 11.841, *p* < 0.01, η^2^ = 0.207 (see Table 3). Additionally, significant age effects were found for all physical parameters (*p* < 0.01; η^2^ ranging from 0.017 to 0.351). In analyzing age categories for physicalperformance, Wilks’ Lambda was Λ = 0.431, F(51, 1316) = 8.430, *p* < 0.01, η^2^ = 0.245.

#### 3.2.3. Effects of Gender

Statistically significant main effects of gender were found across all anthropometric and physiological parameters, except for BMI, fat mass, and flexibility. In the analysis of gender effects on anthropometric measures, Wilks’ Lambda was Λ = 0.657, F(10, 452) = 23.545, *p* < 0.01, η^2^ = 0.343. For physical performance parameters, Wilks’ Lambda for gender effects was Λ = 0.392, F(17, 442) = 40.309, *p* < 0.01, η^2^ = 0.608.

### 3.3. Multiple Regression Models of Physical Performances According to Age Category and Gender

Stepwise regression analysis on performance parameters revealed that body fat percentage, leg muscle volume, standing height, and wingspan were the strongest predictors of physical performance in boys. In contrast, body fat percentage, standing height, and sitting height were the primary predictors for girls. Table 5 presents the regression model outcomes, including the standard error of estimation, with anthropometric variables as independent predictors. These findings highlight the significant role of specific morphological characteristics in predicting and understanding performance variations among athletes (see Table 5).

## 4. Discussion

The objectives of this research were to (i) examine the development of anthropometric characteristics and physical performance among Tunisian basketball athletes across different age categories, and (ii) to identify the variables that best predict performance in jumping ability and speed tests. This study aims to establish reference values for physical performance parameters by gender and age category in Tunisian basketball athletes.

### 4.1. Anthropometry and Physical Performances Differences Based on Age and Gender

The present study demonstrated significant age-related increases in all anthropometric and performance variables. Chronological age and gender influenced morphological (e.g., sitting height, standing height, leg length, body mass, BMI, fat mass, fat-free mass, body fat percentage, wingspan, and leg muscle volume) and physical performance parameters (e.g., VO_2_ max, SJ and CMJ power, jump height, five-jump test, sprint times, agility, and flexibility) across the U15, U17, and U20 age categories. These findings are consistent with research by Aouichaoui et al. [27] on Tunisian handball players and by Tounsi et al. [28] on Tunisian soccer players aged 13 to 19.

The results align with patterns of somatic growth, suggesting that the development of anthropometric and performance parameters may correlate with morphological and hormonal changes associated with peak height velocity (PHV). During pre-puberty, growth is driven by growth hormone (GH) and thyroid hormones (T3 and T4) [29], while maturation is marked by interactions between GH, steroid hormones (especially testosterone and estradiol), gender and insulin-like growth factor I (IGF-I), leading to changes in body composition, including water, muscle, bone, and fat proportions [30]. The average PHV in boys occurs around age 14 [31].

Supporting this, speed and jumping performance progressively improved from the U13 to U20 age groups, with no significant gains observed between U17 and U20. Our findings also indicated that vertical jump and speed performance increase with age, as lower limb performance improves throughout childhood and adolescence [13]. This stage corresponds with anabolic changes in skeletal muscle mass. In boys, this development is further supported by increased circulating testosterone, which induces hypertrophy of type II muscle fibers [32,33].

This study also revealed gender-specific differences in performance, likely due to physiological and biological changes that emerge in boys and girls at the onset of puberty. These differences coincide with increased levels of endogenous hormones, particularly testosterone in boys, which leads to a greater increase in lean muscle mass and volume, enhancing power, strength, and functional capacities. Consistent with this finding, previous research has shown that gender-related differences in physical parameters are minimal at the start of puberty but become more pronounced as hormonal influences take effect [33,34].

### 4.2. Interaction Influence of Age and Gender on Anthropometry and Physical Performances

Our study demonstrated significant interactions between gender and age in relation to anthropometric parameters and fitness tests. Consistent with our findings, Tounsi et al. [28] reported age-related increases in all anthropometric measures among Tunisian adolescents aged 13–19 in both genders. Our results also align with prior studies that observed similar trends, despite differences in the specific physical tests conducted [35].

Variations in anthropometric and physical performance parameters due to growth, development, and gender are likely influenced by differences in muscle mass, hormonal changes, and overall physical maturity. Pre-pubertal athletes generally show less developed anthropometric and physical characteristics compared to those who have completed puberty. Post-puberty, males typically exhibit greater height, wingspan, and muscle mass than females and tend to perform better in strength, speed, and power tests, while females often demonstrate superior flexibility [36,37].

### 4.3. Multiple Regression Models of Physical Performance Outcomes According to Age Category and Gender

A multiple regression model was applied to lower limb jumping measures (SJ height, CMJ height, SJ power, and CMJ power) and sprint parameters (20 m sprint time) across all age groups, analyzing each gender separately. The best prediction model was selected based on the highest determination coefficient (R^2^) across age groups, explaining the variance in the dependent variable. This study established prediction models from a large sample of Tunisian basketball players (n = 469), consistent with prior research on Tunisian athletes aged 7–13, which identified similar predictive parameters [38].

However, our findings diverge from previous studies that highlighted body height as the sole predictor of basketball performance [39,40], and from research suggesting lean body mass as a key predictor of anaerobic performance in basketball [41]. Gryko et al. [26] reported that only the time from PHV predicted 10 m sprint performance in U13-U15 female Polish basketball players.

Few studies provide age- and gender-specific reference values for physical fitness in Tunisian basketball players. Existing studies focus on different athletic populations, such as Lesinski et al. [42], who provided maturity- and gender-specific morphological and physical percentiles across various sports for young German athletes. Other studies have generated physical percentiles for Chinese children and adolescents [43,44]. While similar in offering gender- and age-specific reference values, differences arise due to variations in chronological and biological age, ethnic groups, and the physical characteristics measured.

Rinaldo et al. [45] showed significant correlations between body fat percentage and sprinting/agility performance, with handgrip strength and body mass status associated with upper limb explosive strength in pre-adolescent basketball players. They found that changes in body composition influenced physical performance, especially in speed, agility, and upper limb power [46].

In Tunisian populations, Tounsi et al. [47] provided percentile values for jumping performance in adolescents aged 13 to 19, while Aouichaoui et al. [48] presented percentiles for lower limb jumping in young athletes across various sports. Recently, Aouichaoui et al. [27] established normative values for physical parameters in Tunisian handball players aged 13 to 19. Given the limited literature on age-specific physical fitness reference values for basketball players, our results serve as a foundational resource.

Identifying top basketball talent requires assessing various anthropometric and performance parameters [7]. Our study shows that multiple regression analyses identified body fat percentage, leg muscle volume, standing height, and wingspan as primary predictors of physical performance in boys, while body fat percentage, standing height, and sitting height were the key predictors in girls. Body fat percentage indicates a balance of muscle and body fat, which is vital for optimizing athleticism. Standing and sitting heights, crucial for rebounding and shot-blocking, along with leg muscle volume (important for explosive movements and endurance) and wingspan (beneficial for defense and offense), contribute to well-rounded basketball skills [8,49].

The best parameters for jump performance, used as dependent variables in our regression models, include vertical jump height and relative power in both SJ and CMJ. These metrics indicate body power and explosive strength, critical for rebounding and shooting. Additionally, 20 m sprint times (with and without the ball) were measured, with the types of jumps assessed closely mimicking common sprinting movements in basketball [49].

## 5. Practical Applications

The established reference values can guide individualized training programs tailored to athletes’ physical profiles, age, and gender, helping practitioners and coaches identify high-potential talent and detect deficiencies in specific performance areas, such as strength. These values also enable objective, data-driven comparisons of youth basketball players’ physical performance across different regions or countries. By applying these benchmarks, coaches and researchers can enhance player development, optimize training effectiveness, and deepen their understanding of youth basketball performance.

## 6. Strengths and Limitations

This study provides a comprehensive dataset of age- and gender-specific physical performance benchmarks for Tunisian youth basketball players, enhancing its relevance for coaches, trainers, and sports scientists working with this demographic. By incorporating a broad array of anthropometric and physical metrics, the study offers valuable insights into the developmental patterns across different age categories, assisting in talent identification and personalized training approaches. Additionally, the large sample size contributes to the robustness of the reference values, supporting their applicability across various age and gender groups.

One limitation of this study is its focus on a specific population—Tunisian youth basketball players—which may limit the generalizability of the findings to other populations or regions with different training practices, socio-cultural factors, or genetic backgrounds. Additionally, while the study accounts for basic anthropometric and performance metrics, future research could incorporate more advanced physiological and psychological assessments to provide a holistic view of athletic development. Lastly, as a cross-sectional study, it does not capture longitudinal changes within individuals, which would be valuable for understanding the trajectories of physical performance over time.

## 7. Conclusions

In summary, male athletes outperformed female athletes in both anthropometric and performance variables. This research established age- and gender-specific reference values for physical performance among Tunisian basketball players, providing valuable benchmarks for monitoring the fitness of adolescent athletes. These reference norms enable coaches to objectively assess individual and team performance, understand physical development across age groups, identify talent, and support the structured growth of young athletes.

## Figures and Tables

**Table 1:** Anthropometric measurements of Tunisian basketball players based on age category and gender.

Age Categories (Years)		Under 13	Under 15	Under 17	Under 20
Body mass (kg)	Boys	55.29 ± 13.96	58.32 ± 12.17 *	§ 72.65 ± 13.67 *	78.27 ± 11.97 *
	Girls	52.59 ± 10.49	52.27 ± 9.04 †	† 66.10 ± 13.47	64.91 ± 7.87
Standing height (cm)	Boys	161.49 ± 11.90 *	§ 168.01 ± 11.38 *	§ 181.07 ± 10.96 *	§ 188.12 ± 8.30 *
	Girls	155.90 ± 7.33	†160.64 ± 5.41	† 167.75 ± 6.63	167.96 ± 6.24
Sitting height (cm)	Boys	79.36 ± 5.26	81.48 ± 6.15 *	§ 88.29 ± 5.53 *	90.78 ± 6.40 *
	Girls	77.61 ± 4.14	79.42 ± 4.32 †	†83.66 ± 3.60	85.32 ± 3.95
Leg length (cm)	Boys	82.13 ± 7.62 *	§ 86.52 ± 7.56 *	§ 92.78 ± 7.24 *	§ 97.34 ± 5.61 *
	Girls	78.28 ±4.25	81.73 ± 6.44	83.22 ± 5.22 †	81.70 ± 5.34
Body mass index (kg.m^−2^)	Boys	22.20 ± 4.42	§ 20.39 ± 2.73	§ 21.98 ± 2.97	22.08 ± 2.97
	Girls	21.44 ± 2.54	20.20 ± 3.02	† 23.15 ± 4.18	22.33 ± 2.05
Fat mass (kg)	Boys	6.89 ± 9.61	9.26 ± 4.12	§ 12.27 ± 5.31	11.99 ± 4.48
	Girls	9.85 ± 3.31	8.70 ± 2.63 *	† 12.99 ± 7.41 *	10.94 ± 2.41 *
Fat-free mass (kg)	Boys	48.39 ± 13.00 *	49.05 ± 9.47 *	§ 60.37 ± 10.28 *	§ 66.27 ± 9.15 *
	Girls	42.73 ± 8.18	43.56 ± 6.69 †	51.71 ± 9.05 †	51.49 ± 6.21 †
Body fat percentage (%)	Boys	17.12 ± 5.47	15.53 ±5.09	16.51 ± 4.87 *	15.08 ± 4.15 *
	Girls	17.95 ± 3.33	16.38 ± 2.64 †	18.53 ± 6.56	17.23 ± 1.97
Wingspan (cm)	Boys	168.22 ± 13.13 *	172.80 ± 13.09 *	184.72 ± 11.71 *	§ 192.75 ± 8.59 *
	Girls	159.95 ± 9.79	† 165.14 ± 7.62 *	† 171.12 ± 7.80	170.46 ± 8.03
Leg muscle volume (mL)	Boys	4353.61 ± 1514.22	4648.34 ± 2238.49 *	5738.37 ± 1834.12	5375.74 ± 1759.24
	Girls	3806.47 ± 1707.45	3463.47 ± 1144.77	5353.58 ± 2095.20	4859.78 ± 1957.59

* Significant difference between boys and girls in same age category (*p* < 0.05). § Boys: Bonferroni test age category vs. previous age category (*p* < 0.05). † Girls: Bonferroni test age category vs. previous age category (*p* < 0.05).

**Table 2:** Physical and physiological performance measurements of Tunisian basketball players by age category and gender.

Age Categories (Years)		U13	U15	U17	U20
VO_2_ max (mL.min^−1^.kg^−1^)	Boys	40.32 ± 2.84 *	§ 42.46 ± 3.59 *	§ 44.99 ± 2.97 *	45.44 ± 4.24 *
	Girls	36.73 ± 2.37	36.91± 3.56	† 39.00± 3.85	40.45 ± 3.03
MAV (km.h^−1^)	Boys	11.52 ± 0.8 *	§ 12.13 ± 1.02 *	§ 12.85 ± 0.84 *	12.98 ±1.21 *
	Girls	10.49 ± 0.67	10.57± 0.97	† 11.14 ± 1.10 †	11.56 ± 0.83
SJ height (cm)	Boys	21.03 ± 5.26	§ 24.85 ± 6.17 *	§ 28.98 ± 4.66 *	§ 32.25 ± 5.76 *
	Girls	19.69 ± 3.86	19.76 ± 4.14	20.04 ± 3.85	21.42 ± 3.34
CMJ height (cm)	Boys	22.37 ± 5.48	§ 26.12 ± 6.34 *	§ 29.31 ± 5.07 *	§ 33.87 ± 6.08 *
	Girls	19.73 ± 4.15	20.68 ± 4.15	21.46 ± 4.66	22.62 ± 3.77
SJ power (W.kg^−1^)	Boys	10.75 ± 1.42	§ 12.00 ± 1.76 *	§ 13.38 ± 1.37 *	§ 15.09 ± 2.63 *
	Girls	10.35 ± 1.27	10.32 ± 1.27	10.59 ± 1.36	10.94 ± 0.96
CMJ power (W.kg^−1^)	Boys	11.07 ± 1.42	§12.75 ± 2.14 *	§ 13.96 ± 1.62 *	§ 16.92 ± 3.81 *
	Girls	10.35 ± 1.21	10.95 ± 1.37	10.94 ± 1.34	11.43± 1.34
Five-jump test (m)	Boys	8.57 ± 0.98 *	9.68 ± 1.42 *	11.38 ± 1.20 *	12.14 ± 1.40 *
	Girls	8.04 ± 0.60	† 8.28 ± 0.81	† 8.83 ± 0.78	† 8.76 ± 0.62
5 m sprint time (s)	Boys	1.30 ± 0.19 *	1.23 ± 0.15 *	§ 1.22 ± 0.18 *	1.13 ± 0.14 *
	Girls	1.40 ± 0.15	1.48 ± 0.29 †	1.33 ± 0.18 †	1.29 ± 0.09
10 m sprint time(s)	Boys	2.15 ± 0.30 *	2.06 ± 0.23 *	2.02 ± 0.21 *	1.89± 0.23 *
	Girls	2.66 ± 0.43	2.59 ± 0.44 †	2.26 ± 0.15	† 2.26 ± 0.15
20 m sprint time without the ball (s)	Boys	3.86 ± 0.43 *	3.65 ± 0.40	3.45± 0.23 *	3.30± 0.19 *
	Girls	4.31 ± 0.41	† 4.66 ± 5.09	† 3.89 ± 0.25	3.77 ± 0.22
20 m sprint time with the ball (s)	Boys	4.19 ± 0.42 *	3.95 ± 0.42 *	3.64 ± 0.29 *	3.75 ± 1.22 *
	Girls	4.69 ± 0.39	4.63 ± 0.52	4.22 ± 0.33	4.12 ± 0.26
Agility test (s)	Boys	12.58 ± 0.90 *	12.07 ± 0.42 *	10.82 ± 0.85 *	10.72 ± 0.56 *
	Girls	14.44 ± 1.02	† 13.71 ± 1.07	† 12.83 ± 1.23	12.81 ± 0.91
Flexibility (cm)	Boys	−4.06 ± 8.53	−2.60 ± 8.53	−0.97 ± 7.30	1.55 ± 9.42
	Girls	−3.04 ± 9.53	−1.89 ± 9.63	0.47 ± 9.55	3.47 ± 7.97

* Significant difference between boys and girls in same age category (*p* < 0.05). § Boys: Bonferroni test age category vs. previous age category (*p* < 0.05). † Girls: Bonferroni test age category vs. previous age category (*p* < 0.05). VO_2_ max: maximum oxygen uptake; MAV: Maximum Aerobic Velocity; SJ: squat jump; CMJ: counter-movement jump.

**Table 3:** The results of the MANOVA analyses evaluating the difference in anthropometric variables by age categories and gender.

	MANOVA Age * Gender		MANOVA Age		MANOVA Gender	
	[F (p)]	η^2^	[F (p)]	η^2^	[F (p)]	η^2^
Body mass (kg)	4.413 (0.004) *	0.028	75.028 (0.000) *	0.328	34.604 (0.000) *	0.070
Standing height (cm)	16.329 (0.000) *	0.096	101.951 (0.000) *	0.399	168.163 (0.000) *	0.267
Sitting height (cm)	3.756 (0.011) *	0.024	82.000 (0.000) *	0.348	45.387 (0.000) *	0.090
Leg length (cm)	21.350 (0.000) *	0.122	37.412 (0.000) *	0.196	171.170 (0.000) *	0.271
Body mass index (kg.m^−2^)	1.464 (0.224)	0.009	14.406 (0.000) *	0.086	0.138 (0.710)	0.000
Fat mass (kg)	2.417 (0.066)	0.015	15.555 (0.000) *	0.092	1.005 (0.317)	0.002
Fat-free mass (kg)	7.556 (0.000) *	0.047	65.796 (0.000) *	0.300	91.260 (0.000) *	0.165
Body fat percentage (%)	1.151(0.328) *	0.007	5.528 (0.001) *	0.035	14.835 (0.000) *	0.031
Wingspan (cm)	14.437 (0.000) *	0.086	62.497 (0.000) *	0.289	160.050 (0.000) *	0.258
Leg muscle volume (mL)	1.337(0.262)	0.009	18.408 (0.000) *	0.107	12.611 (0.000) *	0.027

Multivariate F statistics. *: indicates significant results (*p* < 0.05). In case of non-significant multivariate test results, univariate test results were not shown.

**Table 4:** The results of the MANOVA analyses evaluating the difference in physical performance outcomes by age category and gender.

	MANOVA Age * Gender		MANOVA Age		MANOVA Gender	
	[F (p)]	η^2^	[F (p)]	η^2^	[F (p)]	η^2^
VO_2_ max (mL. min^−1^.kg^−1^)	1.570 (0.196)	0.010	34.463 (0.000) *	0.184	202.532 (0.000) *	0.308
MAV (km.h^−1^)	1.557 (0.199)	0.010	35.056 (0.000) *	0.187	206.087 (0.000) *	0.310
SJ height (cm)	17.621 (0.000) *	0.103	33.446 (0.000) *	0.180	178.779 (0.000) *	0.281
CMJ height(cm)	12.944 (0.000) *	0.078	34.886 (0.000) *	0.186	170.922 (0.000) *	0.272
SJ Power (W.kg^−1^)	23.779 (0.000) *	0.135	46.345 (0.000) *	0.233	191.478 (0.000) *	0.295
CMJ Power (W.kg^−1^)	27.727 (0.000) *	0.154	50.489 (0.000) *	0.249	177.259 (0.000) *	0.279
Five-Jump test (m)	34.795 (0.000) *	0.186	82.659 (0.000) *	0.351	330.526 (0.000) *	0.419
5 m sprint time (s)	3.739 (0.011) *	0.024	15.337 (0.000) *	0.091	66.532 (0.000) *	0.127
10 m sprint time(s)	5.970 (0.001) *	0.038	30.961 (0.000) *	0.169	209.629 (0.000) *	0.314
20 m sprint time without the ball (s)	0.587 (0.624)	0.004	2.582 (0.053)	0.017	7.664 (0.006) *	0.016
20 m sprint time with the ball (s)	1.587 (0.192)	0.010	17.435 (0.000) *	0.102	77.614 (0.000) *	0.145
Agility test (s)	1.372 (0.251)	0.009	65.412 (0.000) *	0.300	351.408 (0.000) *	0.434
Flexibility (cm)	0.161 (0.923)	0.001	9.908 (0.000) *	0.061	2.070 (0.151)	0.004

Multivariate F statistics. *: indicates significant results (*p* < 0.05). In case of non-significant multivariate test results, univariate test results were not shown. VO_2_ max: maximum oxygen uptake; MAV: Maximum Aerobic Velocity; SJ: squat jump; CMJ: counter-movement jump.

**Table 5:** Predictive models for the performance of Tunisian basketball players.

Dependent Variable	Age Category	Gender	Predictors	Constant	Beta (95% CI)	R^2^	Adjusted R^2^	SEE	ANOVA
SJ height	U13	Boys	Body fat percentage	31.568	−0.615 (−0.846 to −0.385)	0.408	0.394	4.101	0.000
		Girls	Sitting height	54.132	−0.444 (−0.838 to −0.050)	0.226	0.186	3.485	0.029
	U15	Boys	Leg muscle volume	26.217	0.001 (0.001 to −0.002)	0.361	0.346	4.995	0.000
			Body fat percentage		−0.455 (−0.668 to −0.242)				
		Girls	Fat mass	26.887	−0.818 (−1.124 to −0.512)	0.273	0.254	3.576	0.000
	U17	Boys	Body fat percentage	36.632	−0.463 (−0.712 to −0.215)	0.235	0.218	4.124	0.000
		Girls	Fat-free mass	27.458	−0.143 (−0.256 to −0.031)	0.113	0.096	3.666	0.014
	U20	Boys	Body fat percentage	80.230	−0.866 (−1.133 to −0.599)	0.482	0.455	4.257	0.000
			Standing height		−0.214 (−0.352 to −0.076)				
			Leg muscle volume		0.001 (0.000 to 0.002)				
		Girls	Wingspan	34.551	−0.235 (−0.356 to −0.114)	0.211	0.179	3.032	0.001
			Standing height		0.269 (0.101 to 0.438)				
			Sitting height		−0.216 (−0.420 to −0.011)				
CMJ height	U13	Boys	Body fat percentage	7.873	−0.475 (−0.740 to −0.210)	0.404	0.375	4.337	0.000
			Age		1.855 (0.094 to 3.616)				
		Girls	Sitting height	54.116	−0.443 (−0.875 to −0.010)	0.195	0.152	3.826	0.045
	U15	Boys	Age	27.758	3.040 (0.050 to −6.030)	0.406	0.384	4.981	0.000
			Body fat percentage		−0.422 (−0.875 to −0.010)				
			Leg muscle volume		0.001 (0.000 to 0.002)				
		Girls	Body fat percentage	27.344	−0.765 (−1.080 to −0.450)	0.236	0.226	3.656	0.000
	U17	Boys	Body fat percentage	38.203	−0.538 (−0.802 to −0.274)	0.268	0.252	4.385	0.000
		Girls	Fat mass	24.133	−0.206 (−0.373 to −0.038)	0.107	0.089	4.453	0.017
	U20	Boys	Body fat percentage	81.115	−0.892 (−1.180 to −0.604)	0.458	0.430	4.594	0.000
			Leg muscle volume		0.001 (−0.000 to 0.002)				
			Standing height		−0.211 (−0.360 to −0.063)				
		Girls	Body fat percentage	24.556	0.172 (−1.128 to 1.472)	0.240	0.127	3.531	0.035
			Fat-free mass		−0.113 (−0.466 to −0.241)				
			Fat mass		−0.313 (−1.876 to 1.250)				
SJ power	U13	Boys	Body fat percentage	13.431	−0.156 (−0.221 to −0.091)	0.358	0.343	1.158	0.000
		Girls	Body mass	17.312	0.057 (−0.329 to 0.443)	0.327	0.208	1.135	0.075
			Wingspan		−0.099 (−0.291 to 0.094)				
			YAPHV		−0.033 (−0.472 to 0.405)				
	U15	Boys	Leg muscle volume	12.019	0.000 (0.000 to 0.001)	0.369	0.353	1.421	0.000
			Body fat percentage		−0.116 (−0.176 to −0.055)				
		Girls	Body fat percentage	12.233	−0.222 (−0.330 to −0.113)	0.181	0.170	1.165	0.000
	U17	Boys	Body fat percentage	15.291	−0.116 (−0.192 to −0.039)	0.172	0.153	1.264	0.004
		Girls	Wingspan	8.553	0.045 (−0.053 to 0.142)	0.082	0.062	1.346	0.236
			Leg length		−0.032 (−0.153 to 0.088)				
			Fat-free mass		−0.056 (−0.112 to 0.000)				
	U20	Boys	Body fat percentage	18.714	−0.368 (−0.504 to −0.232)	0.346	0.323	2.165	0.000
			Leg muscle volume		0.000 (0.000 to 0.001)				
		Girls	Wingspan	18.290	−0.043 (−0.069 to −0.018)	0.130	0.118	0.903	0.001
CMJ power	U13	Boys	Body fat percentage	13.577	−0.146 (−0.213 to −0.079)	0.317	0.300	1.189	0.000
		Girls	Body fat percentage	21.346	−0.058 (−0.414 to 0.298)	0.168	0.021	1.189	0.360
			Sitting height		−0.138 (−0.316 to 0.039)				
			Fat mass		0.077 (−0.328 to 0.481)				
	U15	Boys	Age	−11.192	1.436 (0.468 to 2.405)	0.439	0.418	1.636	0.000
			Body fat percentage		−0.175 (−0.249 to −0.101)				
			Wingspan		0.040 (0.009 to 0.071)				
		Girls	Fat mass	12.867	−0.220 (−0.328 to −0.112)	0.177	0.167	1.257	0.000
	U17	Boys	APHV	−35.193	2.360 (1.284 to 3.436)	0.413	0.373	1.288	0.000
			Body fat percentage		−0.122 (−0.200 to −0.045)				
			Sitting height		0.172 (0.049 to −0.269)				
		Girls	Fat mass	11.743	−0.061 (−0.109 to −0.013)	0.115	0.097	1.277	0.013
	U20	Boys	Body fat percentage	12.222	−0.446 (−0.634 to −0.258)	0.384	0.363	3.047	0.000
			Fat-free mass		0.173 (0.087 to 0.258)				
		Girls	Body fat percentage	12.846	0.233 (0.007 to 0.459)	0.081	0.044	1.311	0.097
			Sitting height		−0.042 (−0.118 to 0.035)				
			Fat mass		−0.172 (−0.357 to 0.014)				
20 m sprint time without the ball	U13	Boys	Body fat percentage	6.138	0.042 (0.026 to 0.058)	0.604	0.585	0.278	0.000
			Sitting height		−0.038 (−0.054 to −0.021)				
		Girls	Sitting height	6545	−0.044 (−0.113 to 0.026)	0.160	0.012	0.416	0.385
			Fat mass		−0.022 (−0.096 to 0.052)				
			Fat-free mass		0.032 (−0.006 to 0.070)				
	U15	Boys	Age	9.161	−0.430 (−0.594 to −0.267)	0.410	0.396	0.313	0.000
		Girls	Body fat percentage		0.026 (0.012 to 0.040)				
	U17	Boys	Body fat percentage	4.329	0.026 (0.015 to 0.037)	0.359	0.331	0.189	0.000
			Age		−0.082 (−0.151 to −0.013)				
		Girls	Body mass	3.557	0.008 (0.003 to 0.013)	0.186	0.153	0.232	0.006
			Leg muscle volume		0.000 (0.000 to 0.000)				
	U20	Boys	Fat mass	2.955	0.029 (0.020 to 0.038)	0.420	0.410	0.153	0.000
		Girls	Fat mass	4.324	0.067 (0.033 to 0.100)	0.191	0.159	0.203	0.001
			Body mass		−0.010 (−0.017 to −0.003)				
			Body fat percentage		−0.036 (−0.072 to 0.000)				
20 m sprint time with the ball	U13	Boys	Body fat percentage	6.081	0.042 (0.026 to 0.059)	0.565	0.544	0.288	0.000
			Sitting height		−0.033 (−0.050 to −0.016)				
		Girls	Sitting height	4.767	−0.055 (−0.135 to 0.025)	0.128	0.032	0.391	0.290
			Wingspan		0.026 (−0.008 to −0.060)				
	U15	Boys	Age	8.276	−0.344 (−0.531 to −0.158)	0.294	0.277	0.358	0.000
			Body fat percentage		0.027 (0.011 to 0.043)				
		Girls	Body mass	3.128	0.020 (0.008 to 0.032)	0.216	0.195	0.474	0.000
			YAPHV		−0.349 (−0.570 to −0.129)				
	U17	Boys	Body fat percentage	5.426	0.031 (0.017 to 0.045)	0.402	0.375	0.232	0.000
			Age		−0.144 (−0.228 to −0.060)				
		Girls	Leg length	3.047	0.028 (0.014 to 0.071)	0.128	0.036	0.385	0.272
			Body fat percentage		−0.032 (−0.088 to 0.023)				
	U20	Boys	Body fat percentage	6.092	0.042(0.025 to 0.059)	0.565	0.532	0.292	0.000
			Wingspan		0.000 (−0.011 to 0.010)				
			Sitting height		−0.032 (−0.058 to −0.006)				
		Girls	Fat-free mass	3.741	0.024 (0.009 to 0.038)	0.129	0.106	0.250	0.006
			Body mass		−0.013 (−0.024 to −0.002)				

CI = confidence interval; R^2^ = coefficient of determination; SEE = standard error of estimation; ANOVA = analysis of variance; YAPHV = years from age at peak height velocity; APHV = age at peak height velocity.

## Data Availability

Data is available from the corresponding authors upon reasonable request.
